# Narrowing region for tropical convections in the western North Pacific

**DOI:** 10.1038/s41598-023-28854-z

**Published:** 2023-01-30

**Authors:** Sanghyeon Yun, Namyoung Kang, Chan Joo Jang

**Affiliations:** 1 Department of Geography, Kyungpook National University, Daegu, 41566 South Korea; 2 Department of Oceanography, University of Science and Technology, Daejeon, 34113 South Korea; 3 Ocean Circulation Research Division, Korea Institute of Ocean Science and Technology, Busan, 49111 South Korea

**Keywords:** Projection and prediction, Environmental impact

## Abstract

Considering that the subtropical highs and tropical convections are observed as negative and positive vorticities respectively, the large-scale features of the atmospheric environment can be effectively represented using streamfunctions as defined by the Laplacian. By investigating the geographical patterns of streamfunctions from different modes of environmental variability, this study conceptualizes how the subtropical high expands and the region for tropical convections migrates in the western North Pacific. It is confirmed that, owing to the expansion of the subtropical high, the limited ocean area for tropical convections even bounded by the equator becomes narrower in the “La Niña mode” than that in the “El Niño mode”. This study finds that a warmer environment is likely to further expand the subtropical high to the west, and then the westernmost shift in the region for tropical convections appears in the “warmer La Niña mode”. A linear perspective suggests that every warmer La Niña environment could be one that people have scarcely experienced before.

## Introduction

Tropical convections including tropical cyclones (TCs) cause numerous deaths and considerable damage to the economy^1–3^. The importance of favorable region for tropical convections lies in that the region implies more active TCs. The region for tropical convections under various climate conditions is thus an important concern to scientists as well as the people residing in and around the tropical ocean basins. The primary concern is related to the region in which TCs occur and the direction in which they move. Although a number of studies have investigated the geographical convective environment in association with the El Niño-Southern Oscillation (ENSO), much remains to be understood about the warmer environment for the convective region.

The ENSO shows a global oceanic and atmospheric oscillations with a two-to-seven-year periodicity^4^. This phenomenon indicates the natural variability between the two extremes of El Niño and La Niña, exhibiting opposite sign of the variability to each other. El Niño is the warm phase of the ENSO cycle with an anomalously high sea surface temperature (SST) in the tropical eastern Pacific, weakening the zonal atmospheric circulation (i.e., the Walker circulation)^5^. As the weakened Walker circulation further weakens the zonal SST gradient, a feedback process enhances the El Niño environment. Conversely, the La Niña environment is characterized by a zonally steeper SST gradient in association with a stronger atmospheric circulation. Various conceptual models have been proposed and continuously improved for the evolution of the ENSO status^6–12^. Generally, the SST anomalies are understood as triggering ENSO phases through westerly wind bursts in the atmosphere^13^, propagating equatorial Kelvin waves to the east^14^ and then forming Rossby waves in the north and the south away from the equator^15^. Off-equatorial oceanic Rossby waves favor low-level atmospheric vorticity, which provides favorable conditions for TC activity. The El Niño environment shows a weaker westward expansion of the subtropical high and thus more intensified TCs in the southeastern quadrant of the western North Pacific^16, 17^. An inverse dynamics works for the La Niña environment, where the SST anomaly increases in the western Pacific. As the stronger zonal SST gradient enhances the Walker curculation, the subtropical high expands well to the west, and the region for TC activity moves westwards. However, while the ENSO is natural variability, global warming is considered an external forcing on the global climate system^4^. A globally warmer environment is examined to show a La Niña-like pattern in the western North Pacific by more heating in the western Pacific warm pool and stronger subtropical high at the same time^18–22^. In a warmer environment, TC formations are observed to show westward displacement, and tracks are likely to be closer to the coastal region of the Asian continent than ever^22^.

This study aims to classify the geographical patterns of the atmospheric environment by different levels of ENSO and global ocean warmth and examine how the region for tropical convections migrates in the western North Pacific. In this study, vorticity field is used to examine the climatological response of tropical convections which include tropical depressions as well as intensified TCs. Spatial distribution of positive vorticities is regarded as contributed by the number, intensity, and duration of local convections. Simultaneously, negative vorticities in the tropics could be considered as contributing to the westward expansion of subtropical high. We utilize the streamfunction to concisely understanding complex atmospheric variables. The low-level streamfunction is defined by the spatial integration of low-level rotational winds^23, 24^. In addition, vorticity is understood as the spatial gradient of rotational winds. As long as the relationship between differentiation and integration is consistent, streamfunction effectively represents the overall feature of local vorticities. Here, by employing a variability mode composed of ENSO and global ocean warmth indexes^25^, the corresponding pattern of streamfunction can be distinguished to reveal a large-scale pattern of subtropical high against tropical convections. Then, we compare the patterns of streamfunction through various modes of environmental variability and discuss the rationale for migrating region of tropical convections.

## Eight modes of environmental variability

**Figure 1:**
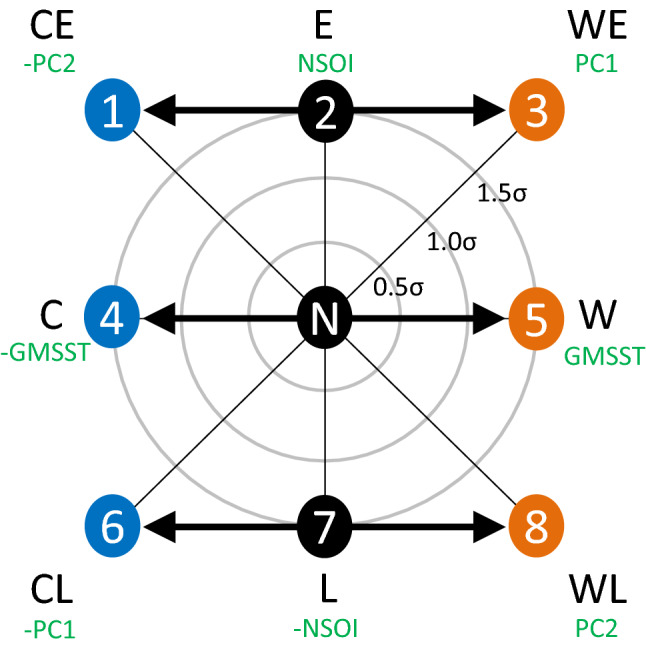
Eight modes of environmental variability. A continuous variability space is formed by the ENSO status (NSOI) and global ocean warmth (GMSST). PC1 and PC2 are the two principal components, which respectively represent the in-phase and out-of-phase modes of GMSST and NSOI. The eight modes of environmental variability are denoted as “Colder El Niño mode (CE)”, “El Niño mode (E)”, “Warmer El Niño mode (WE)”, “Colder mode (C)”, “Warmer mode (W)”, “Colder La Niña mode (CL)”, “La Niña mode (L)”, and “Warmer La Niña mode (WL)” around the “Normal (N)” as respectively numbered from 1 to 8. Three circles indicate 0.5$\sigma$, 1.0$\sigma$, and 1.5$\sigma$ outwards. CE and WE demonstrate the cold and warm anomalies around E, and therefore, 1 and 3 should have values $\sqrt{2}$ times larger than that of E to enable comparison. The same applies for CL and WL as the anomalies around L, and 6 and 8 shows values $\sqrt{2}$ times larger as well.

This study considers climate variability as the directional component of a continuous variability space proposed previously^25, 26^. The variability framework comprises two variables, the negative Southern Oscillation Index (NSOI) and global mean SST (GMSST), which indicate the ENSO status and global ocean warmth, respectively. Here, the “negative” sign for SOI indicates the El Niño variability. Fig. 1 shows the two principal components (PC1 and PC2) from the two primary variables (NSOI and GMSST) on a Cartesian variability space. PC1 is the in-phase mode of NSOI and GMSST, whereas PC2 is the out-of-phase mode of the two. The variance portions of PC1 and PC2 explaining the environment were determined as 54.9 %, and 45.1 %, respectively. In this variability space, three circles indicate 0.5, 1.0, and 1.5 standard deviations ($\sigma$s) outwards, whereas the center is the mean. The continuous variability framework has the merit of showing how environmental variabilities are related to each other. Now, from the variability spectrum, eight variability modes are suggested for environmental guidance. We denote the eight modes as “Colder El Niño mode (CE)”, “El Niño mode (E)”, “Warmer El Niño mode (WE)”, “Colder mode (C)”, ‘Warmer mode (W)’, “Colder La Niña mode (CL)”, “La Niña mode (L)”, and “Warmer La Niña mode (WL)” around the “Normal (N)” as respectively numbered from 1 to 8. In this study, 1.5$\sigma$ levels are used for comparing the atmospheric patterns of E, L, C, and W. Theoretically from a normal distribution, an event at 1.5$\sigma$ level is comparable to the case occurring every 15 years. It is noted that while C and W are the anomalies around N, the anomalies around E are denoted by CE and WE with larger $\sigma$ levels. The same for CL and WL around L. For the anomalies around E and L, 1.5$\sqrt{2}$ $\sigma$ levels are used for comparison.

The ENSO diversity, such as the Central Pacific (CP) ENSO is not explicitly dealt with in this study, but the Niño4 SST which is generally used for indicating CP ENSO, shows that the variability direction for CP El Niño is located between E and WE, whereas that of CP La Niña is identified between CL and L (Supplementary Fig. 1).

## Utilization of streamfunction

This study uses the streamfunction at 850 hPa in investigating the atmospheric patterns corresponding to the eight modes of environmental variability described earlier. The activity of all cyclones in the tropics including tropical depressions as well as intensified TCs is assumed to be printed in the vorticities via the number, intensity, and duration of local convections. The streamfunction is a useful variable for effectively understanding the large-scale pattern of local vorticities, and thus the region for tropical convections.

**Figure 2:**
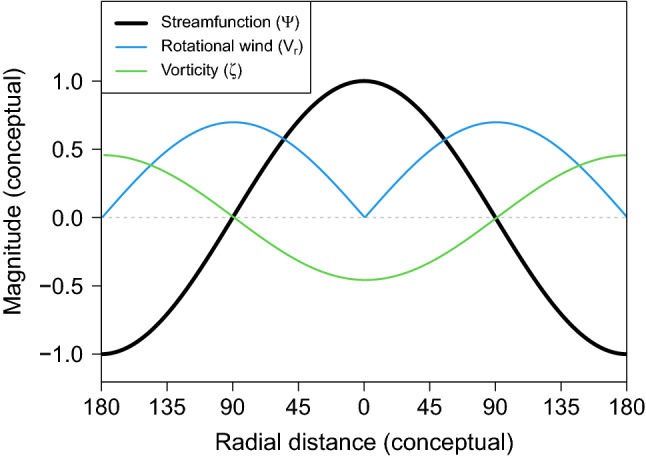
Funcionality of streamfunction. The schematic shows the interrelationship among streamfunction ($\psi$), rotational wind ($V_{r}$), and vorticity ($\zeta$) using a one-dimensional distribution of $\psi$. The distribution of $\psi$ follows a sine curve for example. $V_{r}$ is defined by the gradient of $\psi$, and $\zeta$ is formed by the gradient of $V_{r}$. From the Laplacian, $\psi$ is negatively proportional to $\zeta$, and represents a large-scale pattern of $\zeta$. The value on the x-axis conceptually represents the radial distance from the $\psi$’s maximum. For comparison in the same plot, $V_{r}$ and $\zeta$ are 40 and 1500 times multiplied, respectively.

It is started by decomposing an observed scalar wind (V) into a divergent component ($V_d$) and a rotational component ($V_r$)^23, 24, 27^. Fig. 2 schematically shows the interrelationship among the streamfunction ($\psi$), rotational wind ($V_r$), and vorticity ($\zeta$) using a one-dimensional distribution of $\psi$. Here, a sine wave is suggested as an example of the distribution of $\psi$. The value on the x-axis conceptually represents the radial distance from the maximum of $\psi$. The magnitudes of $V_r$ and $\zeta$ are 40 and 1500 times multiplied, respectively, to compare the shapes of $\psi$, $V_r$ and $\zeta$ at the same time. Mathematically, $V_r$ is defined by the gradient of $\psi$, and $\zeta$ is formed by the gradient of $V_r$. Then, $\zeta$ is simply understood as the Laplacian of $\psi$, i.e., $\nabla ^2 \psi$ = $\zeta$, revealing that $\psi$ is negatively proportional to $\zeta$. The pattern of $\psi$ looks like a virtual hill with slopes from the minimum of $\psi$ to the maximum delineating the large-scale pattern of the subtropical high. The rotational flows (i.e., streamlines) are perpendicular to the slopes, and $\zeta$ is caused by horizontal wind shear. Notably, while local $\zeta$s are determined by the different $V_r$s between the parallel flows, $\psi$ depicts the large-scale pattern of overall $\zeta$s around the maximum or minimum of $\psi$, where the direction of $V_r$ reverses.

## Geographical patterns of streamfunction

**Figure 3:**
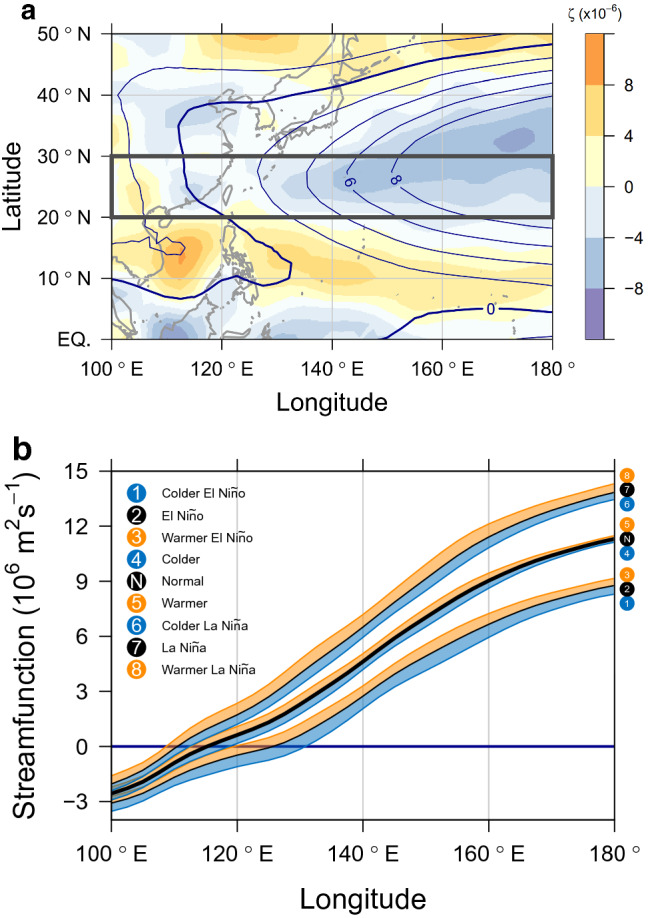
Geographical patterns of streamfunction. (**a**) Mean fields of streamfunction ($\psi$) and vorticity ($\zeta$), and (**b**) the sequential patterns of $\psi$ corresponding to the eight modes of variability. All values are originally calculated in a global domain and zoomed in for the western North Pacific. In (a), local vorticities are shaded following the color legend on the right side, and streamlines are contoured at 10$^6$ m$^2$ s$^{-1}$ intervals. A thick dark blue line indicates a streamline with zero values. The lines in (b) show each longitudinal distribution of $\psi$ averaged over the latitudes between 20$^\circ$ and 30$^\circ$N as denoted by a black rectangle in (a). The thick black line represents the zonal distribution of the mean field as a reference, and the two thin black lines show the ENSO anomalies. Orange and blue shades represent the warm and cold anomalies, respectively. It is found that the patterns are sequentially continuous showing a geographical spectrum, which implies the narrowing region for tropical convections as the chain number moves from 1 to 8.

**Figure 4:**
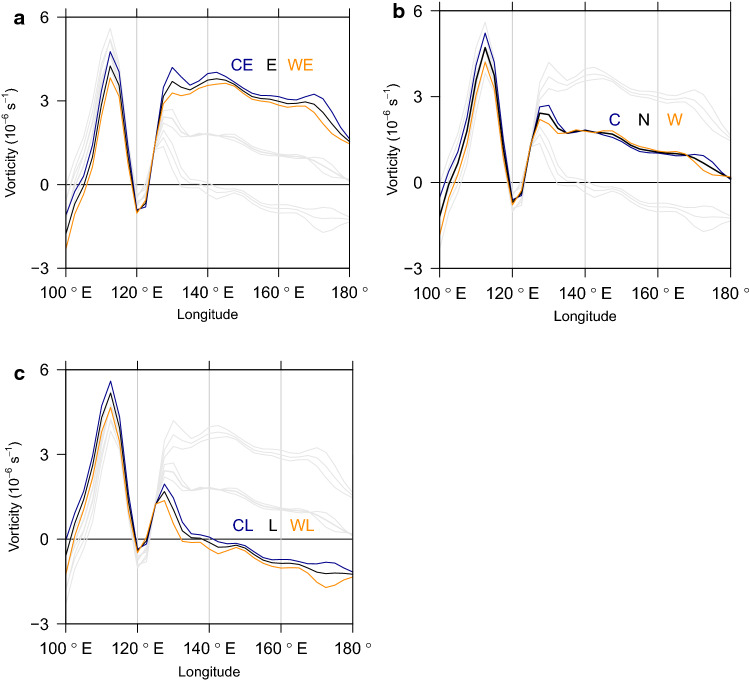
Longitudinal distribution of vorticity. The local vorticities between 5$^\circ$–20$^\circ$N are averaged along 100$^\circ$E–180$^\circ$ for (**a**) “El Niño mode (E) and its anomaly modes (CE, WE)”, (**b**) “Colder mode and Warmer mode (C, W)” around Nomral (N), and (**c**) “La Niña mode and its anomaly modes (CL, WL)”. The black lines from top to bottom represent E, N, and L, and the blue and red lines in each panel show the colder and the warmer anomalies, respectively.

Fig. 3a shows the mean fields of $\zeta$ and $\psi$ from June to November (JJASON) over a 36-year period (1985–2020) at a low-level atmosphere (850 hPa), corresponding to N in Fig. 1. A low level is considered to include even weak tropical convections. These are originally calculated in the global region (Supplementary Figs. 2, 3, 4), and the western North Pacific is zoomed in. Streamlines with positive $\psi$ exhibit a large-scale anticyclonic flow. Then, $\psi$’s pattern shows how far the subtropical high expands to the west during boreal summer and fall (i.e., JJASON). Local positive vorticities mostly appear on the southwestern edge of the subtropical high, and the region with the strongest vorticity is found in the South China Sea.

Now, $\psi$ on every grid point is modeled by the eight modes of environmental variability using the ordinary least squares method (Supplementary Figs. 2, 3, 4). As depicted in Fig. 1, 1.5$\sigma$ levels are used for E, L, C, and W, whereas 1.5$\sqrt{2}$ levels are used for CE, WE, CL, and WL. Fig. 3b shows each longitudinal distribution of $\psi$ averaged over the latitudes between 20$^\circ$ and 30$^\circ$N (see the black rectangle in Fig. 3a). By investigating the geographical patterns of $\psi$ from different modes of environmental variability, this study conceptualizes how the subtropical high expands and the region for tropical convections migrates in the western North Pacific. It is found that the patterns are sequentially continuous, showing a geographical spectrum. The $\psi$’s difference between the ENSO anomalies around N is most apparent. C and W modes provide opposite anomalous patterns around N. The colder and warmer environments of the ENSO anomalies even make a spectrum of $\psi$’s distribution. The slope of $\psi$ confirms that the center of the subtropical high is located in the east and the region for tropical convections lies in the west. The ocean area for tropical convections is geographically limited by the Indochina Peninsula and the Maritime Continent. When the Coriolis force by latitude is taken into account, the area should be even bounded by the equator where the vorticity sign changes. Under these circumstances, the expansion of $\psi$ would directly imply a narrower region for tropical convections. It is confirmed that the W mode (as well as the L mode) is likely to provide a larger area of subtropical high than N. The WL mode is noticeable among the patterns because it shows the farthest expansion of the subtropical high to the west, implying the narrowest region for tropical convections.

**Figure 5:**
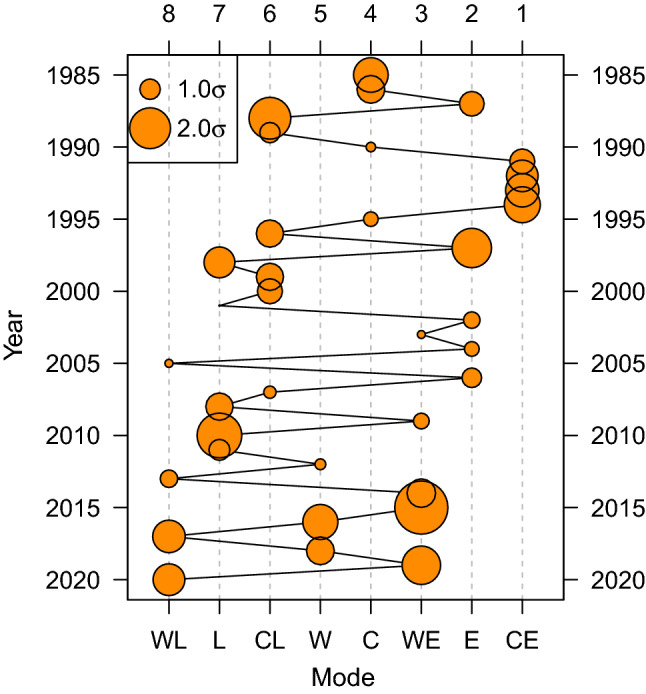
Chronology of the modes of environmental variability. For each year, the closest variability direction among the eight modes is shown with its magnitude. A smaller circle implies that the variability is less anomalous and closer to N. El Niño and La Niña trends repeat over time and tilt toward each warmer mode such as WE and WL. Considering the westernmost expansion of the atmospheric pattern in WL, a linear perspective suggests that every warmer La Niña environment could be one that people have scarcely experienced before.

Narrowing region for tropical convections according to expanding subtropical highs is confirmed by Fig. 4. The latitudinal area (5$^\circ$–20$^\circ$N) is designed to examine how the main region for TC activity responds to the expanding subtropical highs. Vorticity maximum in the South China Sea and its reduction over the land area of the Philippines appear in every mode of variability. E mode and its anomalies (CE, WE) in (a) show the largest area of positive vorticity, and the vorticity maximum in the Philippine Sea looks nearly comparable to the South China Sea. On the other hand, L and its anomalies (CL, WL) in (c) experience a clear reduction of local vorticities in most areas. The vorticity sign even changes to negative (140$^\circ$E–180$^\circ$), except for some small area to the east of the Philippines. Some area of increasing vorticity also appears in the west of the South China Sea. It is noticeable that all three warm anomalies (WE, W, WL) around E, N, L exhibit weaker vorticities than the cold opponents (CE, C, CL) over the whole region in the western North Pacific. This reflects the geographical spectrum of the atmospheric patterns corresponding to the modes of variability (see Supplementary Fig. 5 for significance test).

## Chronology of the modes of environmental variability

Based on the continuous variability framework shown in Fig. 1, an annual event can be identified as one of the eight modes of environmental variability (Supplementary Fig. 7). For each year, the closest variability direction among the eight modes is shown in Fig. 4. In the abscissa, the names of the modes are denoted in the order of CE, E, WE, C, W, CL, L, and WL from right to left, in accordance with the westward expanding subtropical highs. The size of the circles represents the magnitude in each variability direction. A smaller circle implies that the variability is less anomalous and closer to N. In association with the atmospheric patterns (see Fig. 3) and the variable region for tropical convections (see Figs. 4), 5 takes the role of instruction manual for understanding which pattern a certain year belongs to and how strong it was. The chronology of the variability modes exhibits that El Niño and La Niña trends repeat over time and tilt toward each warmer mode such as WE and WL. Considering the westernmost pattern of WL, among others, a linear perspective suggests that every warmer La Niña environment could be one that people has scarcely been experienced before.

## Summary and discussion

By investigating the geographical patterns of streamfunctions from different modes of environmental variability, this study conceptualizes how the subtropical high expands and the region for tropical convections migrates in the western North Pacific. In this study, positive vorticities are regarded as representing the tropical convections which include tropical depressions as well as intensified TCs. The research period is set to 36 years from 1985 to 2020, and all variables are averaged over the six months (i.e., JJASON) when tropical convections are relatively active in the western North Pacific. The year 1985 comes from the previous work^28^ about the reliable consensus on TC climatologies between the best-track data from operational agencies. Though not explicitly dealing with observed TCs, the study results could help with understanding TC activity in the previous and future studies. First, a continuous variability space^25^ is used to assign the eight modes for directional variabilities. This variability framework includes two environmental indexes for the ENSO status and global ocean warmth, and the eight modes of environmental variability are named CE, E, WE, C, W, CL, L, and WL. E and L denote the anomalous modes around N when considering opposite variability directions, i.e., the El Niño and La Niña, respectively. C and W indicate cold and warm anomalies, and their combinations are represented as CE, WE, CL, and WL. Then, their corresponding streamfunctions at a low-level atmosphere (850 hPa) are investigated to find the large-scale pattern of the local vorticities as explained by the Laplacian explains. The patterns are sequentially continuous, and exhibit a geographical spectrum. A greater expansion of the subtropical high is observed in the L mode than that in the E mode. A warmer environment is likely to further expand the subtropical high, and thus the WL mode exhibits the highest expansion, implying the narrowest region for tropical convections. The chronology of the variability modes shows that the El Niño and La Niña trends repeat over time and tilt toward each warmer mode. A linear perspective suggests that every warmer La Niña environment could be one that people have scarcely experienced before.

The results could be exploited in conceptualizing the region for tropical convections given the levels of ENSO status and global ocean warmth. The value of this study lies in that the large-scale features of low-level vorticities are concisely classified into the eight patterns of streamfunction. Tropical cyclone activities, whether weak or strong, are assumed to contribute to the local vorticities, and this study uses streamfunction as a proxy for evaluating the large-scale inhibition of TC activity. Future research may involve operational TC best-track data and examine how the localized TC activity and its attributes such as the local frequency, intensity, and speed are geographically distributed by each mode of variability. In addition, while the current study deals with a climatological perspective on the observed atmospheric patterns only in the western North Pacific, future studies could extend the research scope to inter-basin relationship and its associated SST patterns^29, 30^.

## Supplementary Information

Supplementary Information.

## Data Availability

The Southern Oscillation Index (SOI) from the National Oceanic and Atmospheric Administration (NOAA)/Climate Prediction Center (http://cpc.ncep.noaa.gov/data/indices/soi) is used For ENSO status. Global ocean warmth is indicated by global mean SST (GMSST) coming from Extended reconstructed SST version 5^31^ of the NOAA/National Centers for Environmental Prediction (NCEP) reanalysis (http://esrl.noaa.gov/psd/data/gridded). The zonal winds and meridional winds from the National Oceanic and Atmospheric Administration (NOAA)/National Centers for Environmental Prediction (NCEP) reanalysis (https://psl.noaa.gov/cgi-bin/data/timeseries/timeseries1.pl) are used to calculate the streamfunction. The research period is set as 36 years from 1985 to 2020. Values for all variables are averaged over the months from June to November (JJASON). All the analyses and figures are created using the software R (http://r-project.org) and are available online (https://www.rpubs.com/yunsh823/P2022b). The datasets for the analyses and figures are available from the corresponding author on reasonable request.
