# Supplementary material for: Narrowing region for tropical convections in the western North Pacific
Source: Sci Rep. 2023 Jan 30;13:1664. doi: 10.1038/s41598-023-28854-z (PMC9886944; doi:10.1038/s41598-023-28854-z)
Supplement: Supplementary file 1 — Supplementary Information. [file 41598_2023_28854_MOESM1_ESM.pdf]

Supplementary Information

1 Eight modes of environmental variability and the variability direction of CP ENSO.

This study represents ENSO only by SOI. and does not explicitly deal with a variant such as CP ENSO. On the other hand, the research framework in Supplementary Fig. 1 identifies the variability direction of CP ENSO indexed by NINO4 SST. NINO4 SST shows the highest correlation {r = +0.94; [0.88, 0.97], 95% confidence interval} with a variability between E and WE. A directional variability can be defined by GMSST·cosθ+NSOI·sinθ, where a counterclockwise θ starts from the variability direction of GMSST. The highest correlation coefficient means that the projection length of 1.0σ CP El Niño onto the closest variability appears 0.94σ. While CP El Niño is understood as a variability between E and WE in the research framework, a variability closest to CP La Niña is found between CL and L.

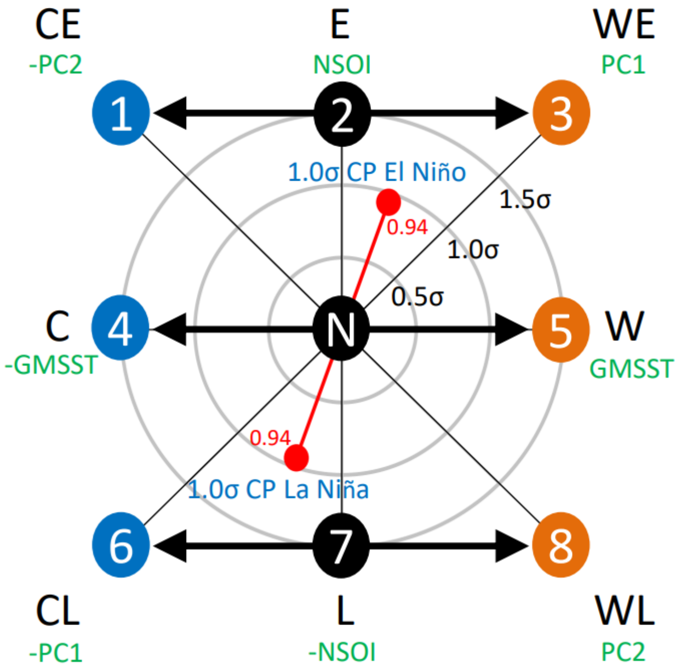

Suppl. Fig. 1. Eight modes of environmental variability and the variability direction of CP ENSO.

2 Atmospheric variables and the Laplacian

Atmospheric variables such as streamfunction (ψ), rotational wind (V_r), and vorticity ($\boldsymbol{\zeta}$) during JJASON over the 36-yr period (1985–2020) at a low-level atmosphere (850 hPa) are modeled by the eight modes of variability (Supplementary Fig. 2–4). The Laplacian explains that V_r is the spatial integration of local $\boldsymbol{\zeta}$s, and ψ is the spatial integration of local V_rs. Then, ψ is considered as showing a large-scale feature of local $\boldsymbol{\zeta}$s. The differences among the modes of variability may not be easily captured in these global maps, but the maps for western North Pacific only (Fig. 3 in the main manuscript, and Supplementary Fig. 5) clearly shows a sequentially expanding subtropical highs along the chain number from 1 to 8.

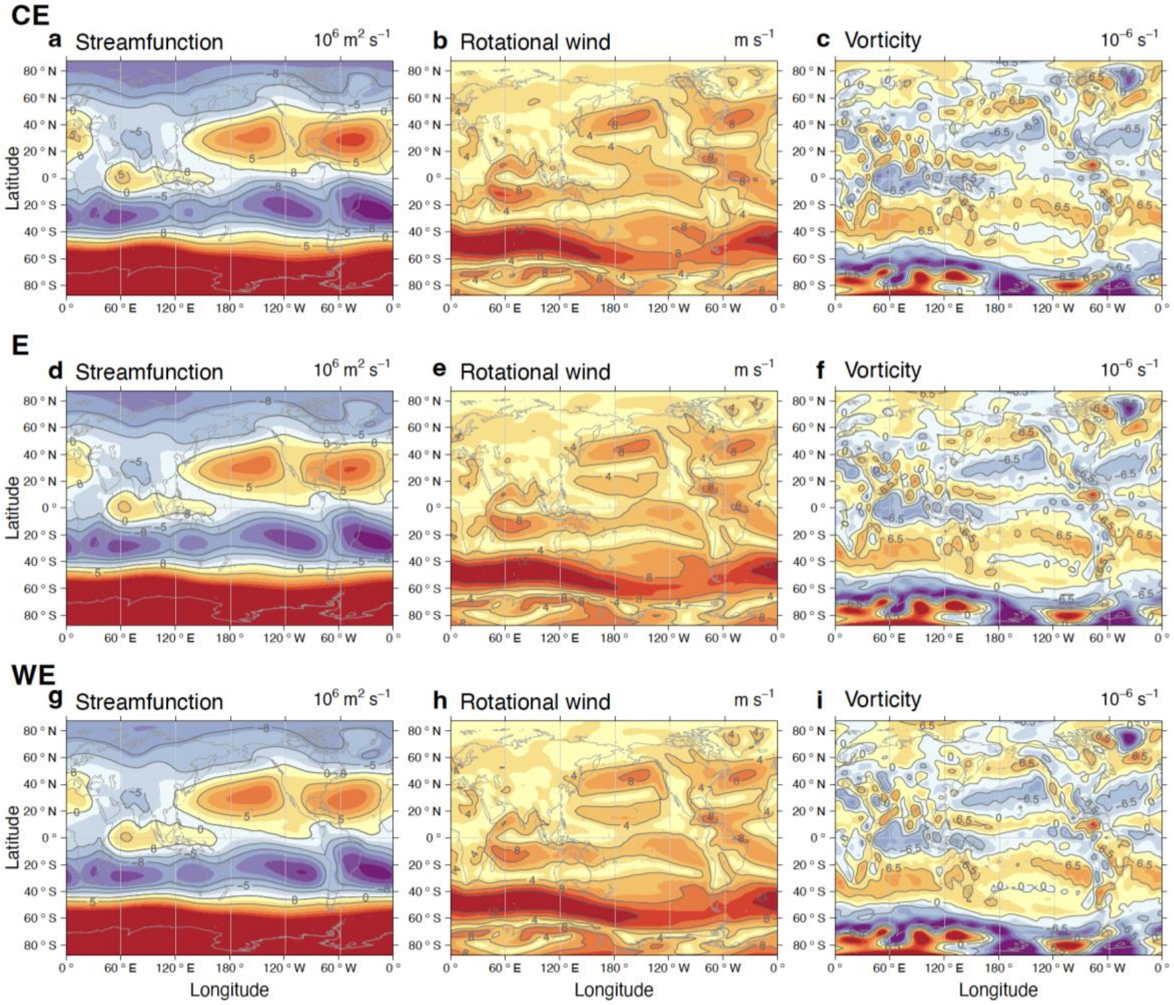

Suppl. Fig. 2. Modeled atmospheric patterns corresponding to “El Niño mode (E)” and its anomaly modes. The first row **(a–c)** exhibits streamfunction, rotational wind, and vorticity for “Colder El Niño mode (CE)”. The second row **(d–f)** shows the same attributes for “Colder El Niño mode (CE)” and the third row **(g–i)** for “Warmer El Niño mode (WE)”.

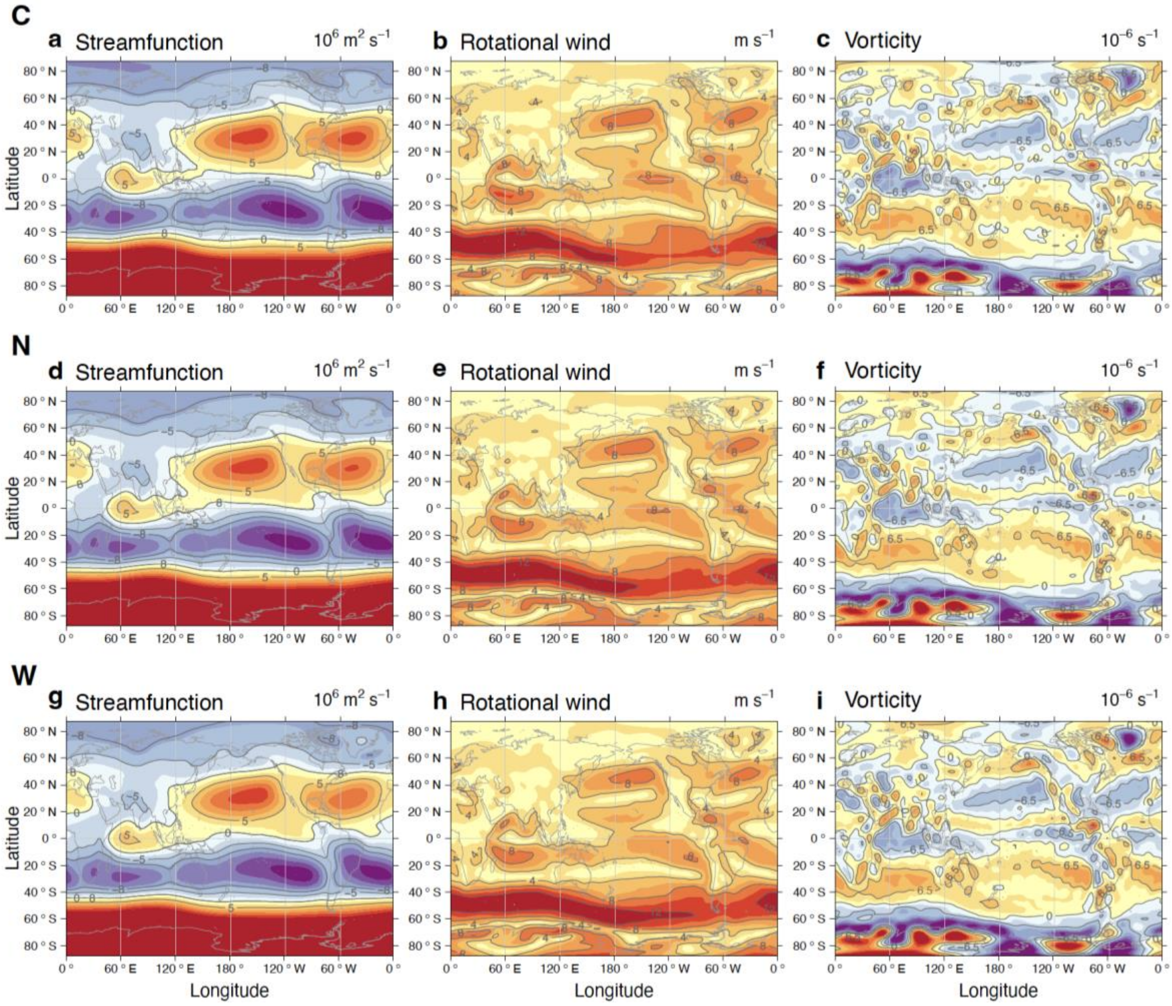

Suppl. Fig. 3. The same as in Supplementary Fig. 2, but for "Colder mode (C)", "Normal (N)", and "Warmer mode (W)" from top to bottom.

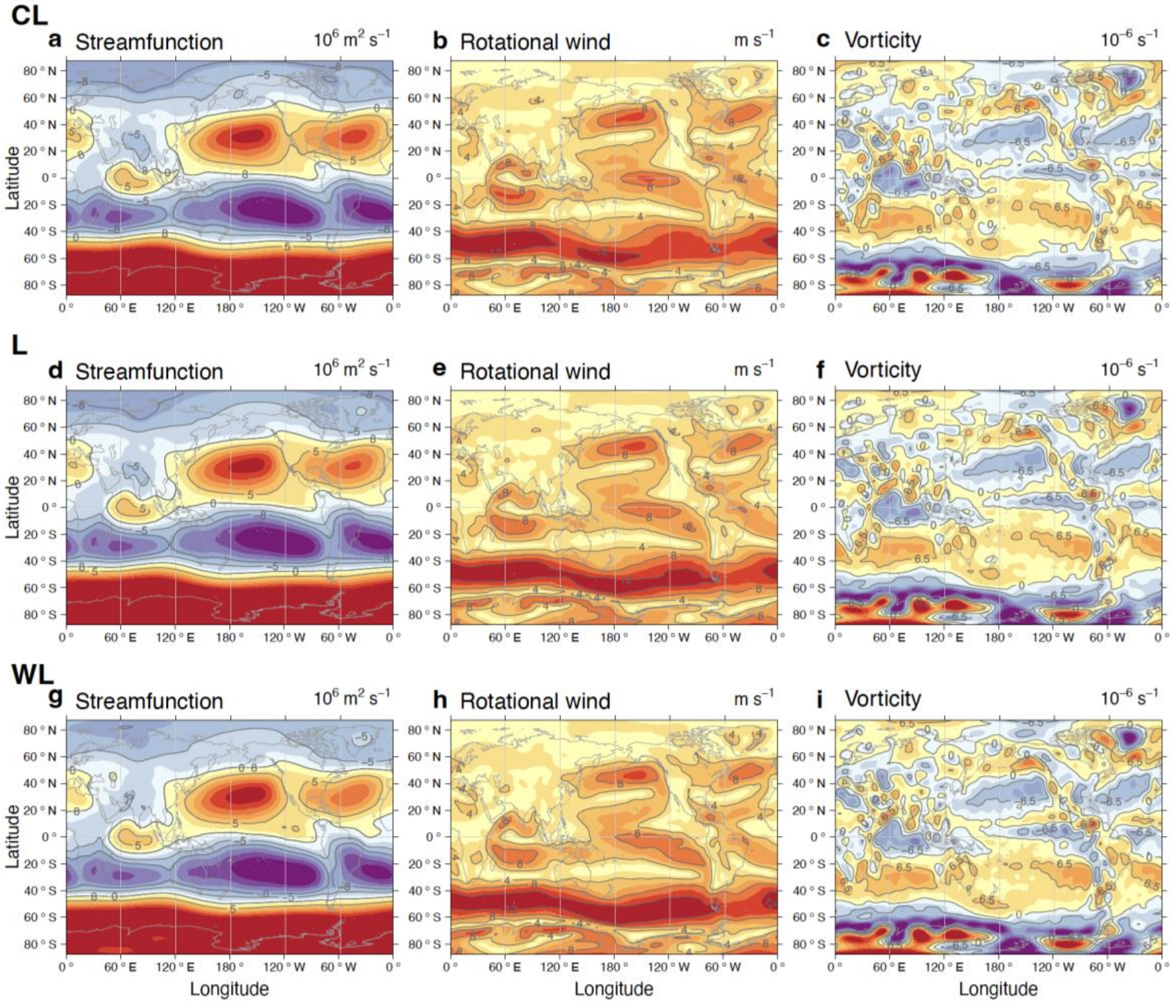

Suppl. Fig. 4. The same as in Supplementary Fig. 2, but for "Colder La Niña mode (CL)", "La Niña mode (L)", and "Warmer La Niña mode (WL)" from top to bottom.

3 Geographical spectrum of streamfunction

Supplementary Fig. 5 demonstrates the streamfunction for each mode of variability. The expansion of subtropical high between CE to LW is most apparent, and the geographical spectrum of the patterns is shown in Fig. 3b (main manuscript). Blue crosses demonstrate the significant area at 95% confidence level. It shows that significant areas differ by modes of variability. Here in (a) and (h), we find that CE and WL modes show significant response in most areas, which support the conclusion of the current study, that is “This study finds that a warmer environment is likely to further expand the subtropical high to the west, and then the westernmost shift in the region for tropical cyclone activity appears in the “warmer La Niña mode” (in main manuscript).

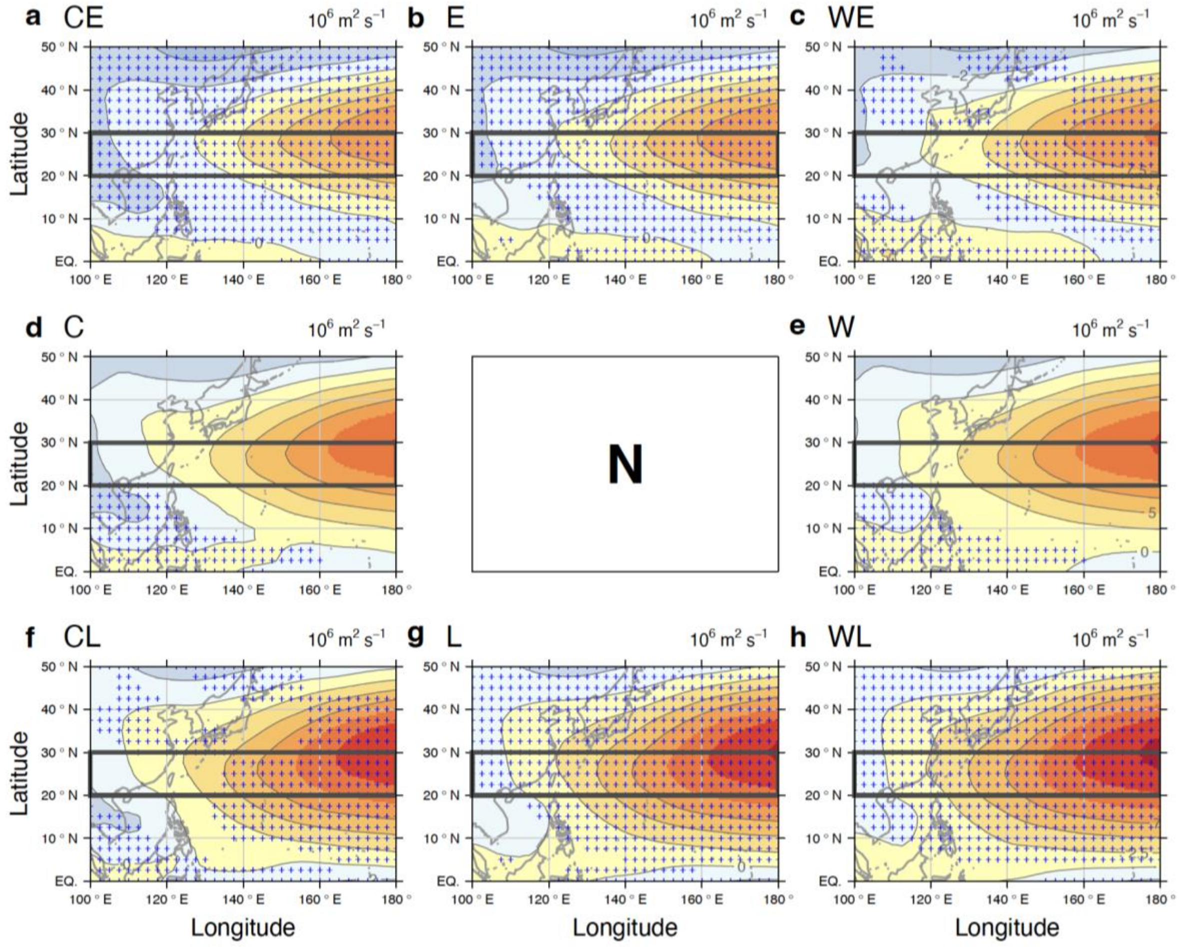

Suppl. Fig. 5. Modeled streamfunction for each mode of variability, and the significant area at 95 % confidence level denoted by blue crosses. The black rectangles shown are the same as in Fig. 3 (main manuscript).

4 Density distribution of TC genesis longitude

Narrowing region for TC activity according to expanding subtropical highs is also examined by Supplementary Fig. 6. The results generally show the similar responses as vorticities in Fig. 4 (main manuscript), but the response looks poor in describing the patterns compared to the vorticities. The westward moving pattern appears mostly clear in (d). In (a), the pattern of WE slightly shows westward difference from CE, but not apparent. The patterns between C and W in (c) are not consistent with Fig. 4. Only with the limited number of events (i.e., MSW >= 17 m/s), the density distribution may not realistically delineate the patterns corresponding to the modes of environmental variability. Observations come from the Join Typhoon Warning Center (JTWC; http://www.usno.navy.mil/NOOC/nmfc-ph/RSS/jtwc/best_tracks) best-track data over the 36 years (1985-2020) during JJASON.

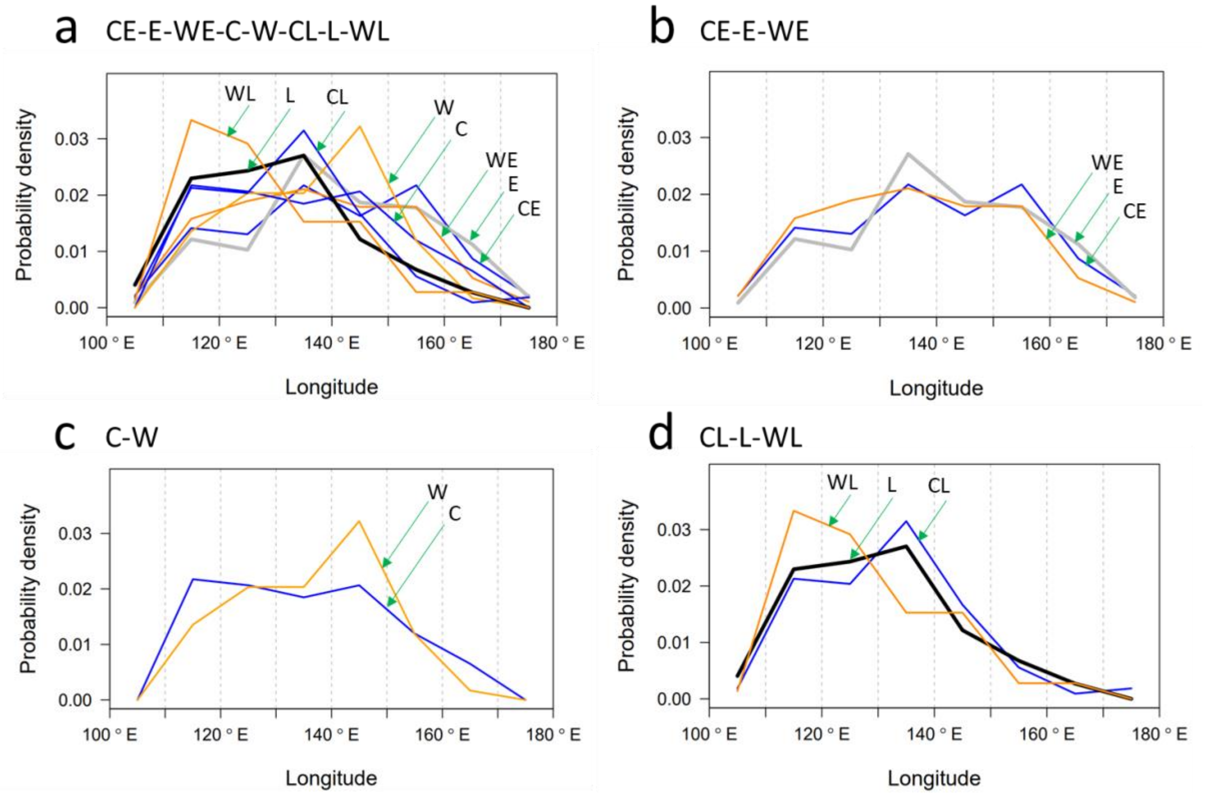

Suppl. Fig. 6. Density distribution of TC genesis longitude. The longitudinal densities of TCs occurred within 5º–20ºN and 100ºE–175ºE are shown for **(a)** all modes, (b) “El Niño mode (E) and its anomaly modes (CE, WE)”, **(c)** “Colder and Warmer modes (C, W)”, and **(d)** “La Niña mode (E) and its anomaly modes (CL, WL)”. Observations come from the Join Typhoon Warning Center (JTWC) best-track data over the 36 years (1985-2020) during JJASON.

5 Assigning the closest variability mode to annual observation

In a Cartesian space, each observation indicates the ENSO status and the level of global ocean warmth for JJASON per year (Supplementary Fig. 7). There are 36 observations (1985–2020) on an annual basis. The closest variability mode among the eight is assigned to each year. While black color represents the events closest to ENSO modes, blue and orange around denote the events closest to the colder anomalies (CE, CL) and the warmer anomalies (WE, WL), respectively. The rest (C, W) are colored in green.

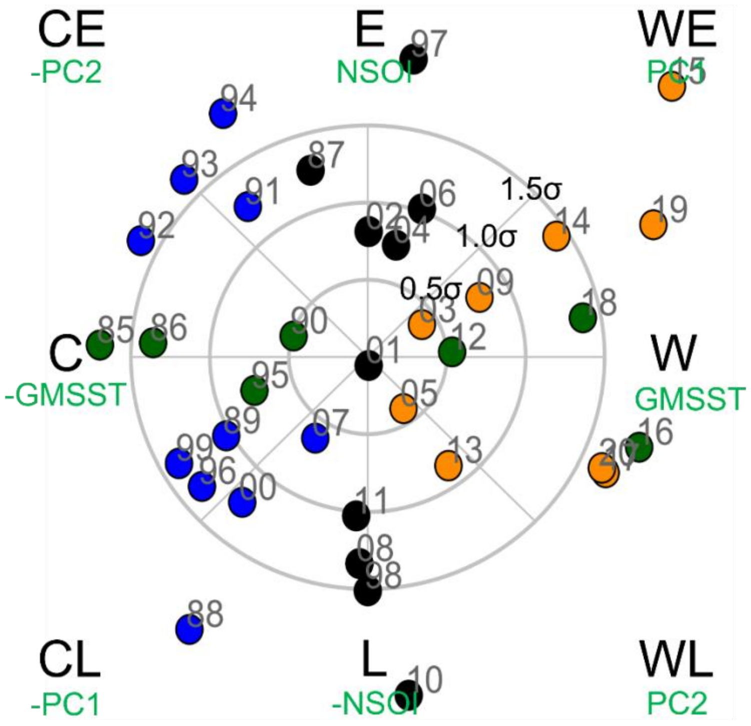

Suppl. Fig. 7. Observed ENSO status and global warmth level over the 36 years (1985–2020) during JJASON. Each circle indicates the closest mode of environmental variability by different colors. Cold and warm anomalies are denoted by blue and orange color, respectively. The rest (C, W) are colored in green. The last two digits of year number are displayed for each year.

6 Continuous variability space

A two-dimensional continuous variability space can be organized using any two primary variables[1]. In the current study, the two variables of NSOI and GMSST are used for the primary variables. The primary variables indicate the time series of annual ENSO status and global warmth level, respectively. Though the two are independent, it doesn't mean they have an orthogonal relationship. Only the principal components are orthogonal meaning no correlation. From the continuous variability space, any directional variability (DV) can be identified as the combination of ENSO status and global warmth level, implying varying weights on the two primary variables. DV can be defined by the angle (θ) starting from GMSST. Here, PC1 and PC2 are understood as special cases when equal weights are applied to the primary variables. Equal weights mean $\theta = \pi/4$. While PC1 is the directional variability where equal weights are given to NSOI and GMSST, while PC2 is the same as PC1 but with negative sign on NSOI. This method attempts to show that the variabilities are continuously linked and so do the patterns by the modes of environmental variability.

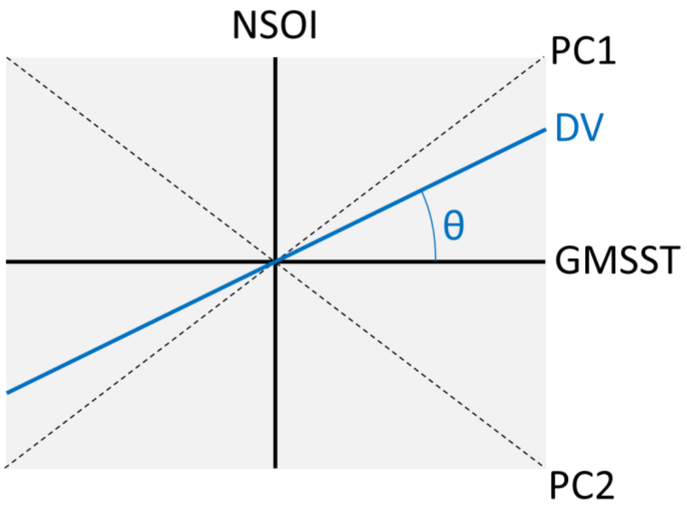

Suppl. Fig. 8. A diagram of two-dimensional continuous variability space. The two variables of NSOI and GMSST are used for the primary variables. PC1 and PC2 represent the two principal components. DV denotes a directional variability depending on a counterclockwise angle (θ) starting from GMSST.

---

[1] Kang, N., Elsner, J.B.: An empirical framework for tropical cyclone climatology. Clim. Dyn. **39**, 669–680 (2012). https://doi.org/10.1007/s00382-011-1231-x
